# Supplementary material for: Whole-genome sequencing of Schistosoma mansoni reveals extensive diversity with limited selection despite mass drug administration
Source: Nat Commun. 2021 Aug 6;12:4776. doi: 10.1038/s41467-021-24958-0 (PMC8346512; doi:10.1038/s41467-021-24958-0)
Supplement: Supplementary file 1 — Supplementary Information [file 41467_2021_24958_MOESM1_ESM.pdf]

SUPPLEMENTARY INFORMATION

Whole-genome sequencing of *Schistosoma mansoni* reveals extensive diversity with limited selection despite mass drug administration

Duncan J. Berger, Thomas Crellen, Poppy H. L. Lamberton, Fiona Allan, Alan Tracey, Jennifer D. Noonan, Narcis B. Kabatereine, Edridah M. Tukahebwa, Moses Adriko, Nancy Holroyd, Joanne P. Webster, Matthew Berriman, James A. Cotton

Supplementary Figures 1-19

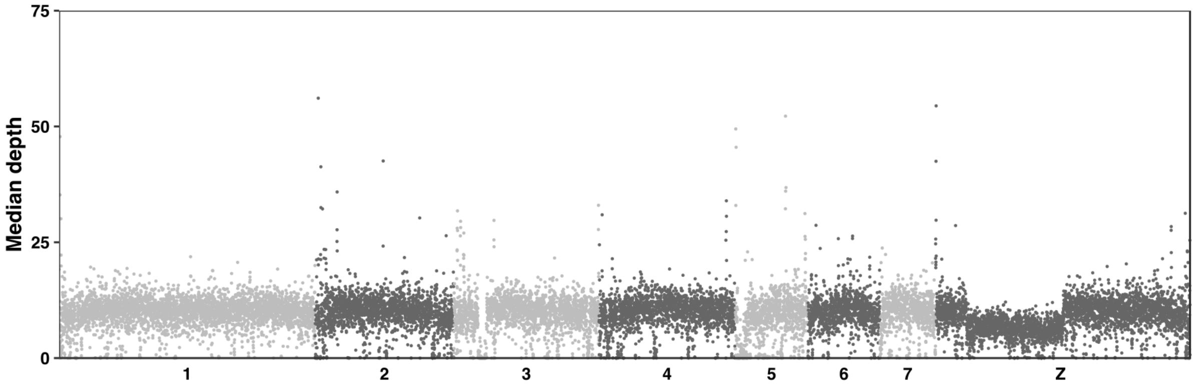

Supplementary Figure 1: Depth of read coverage. Median chromosomal coverage across 198 miracidia samples. Coverage was calculated in non-overlapping 25 kb windows and median values for each window across all samples was plotted across all chromosomes (shown in alternating shades of grey).

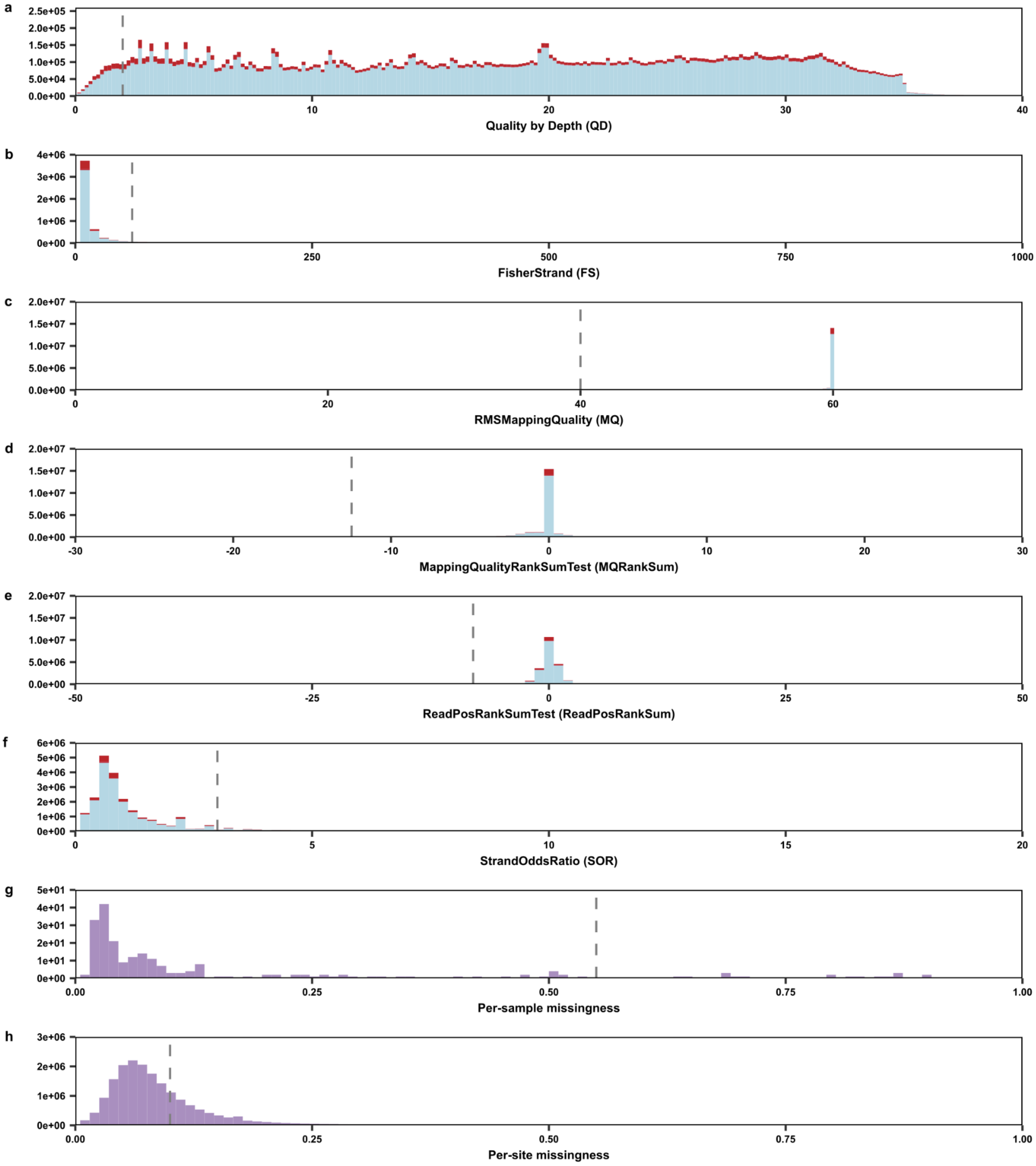

Supplementary Figure 2: Variant quality control. Plots a-f show the frequency distribution of annotation values for 17,395,313 single nucleotide polymorphisms (SNPs) (light blue), indels and mixed sites (SNPs and indels called at the same position for different samples) (red). Plots g&h show the frequency distribution of per-sample missingness (samples with a high rate of per-site variant missingness) and per-site (sites with a high proportion of variant missingness) missingness after filtering using the thresholds in a-f for all remaining variants (purple). Vertical dashed lines show the thresholds applied in the study for removing sites.

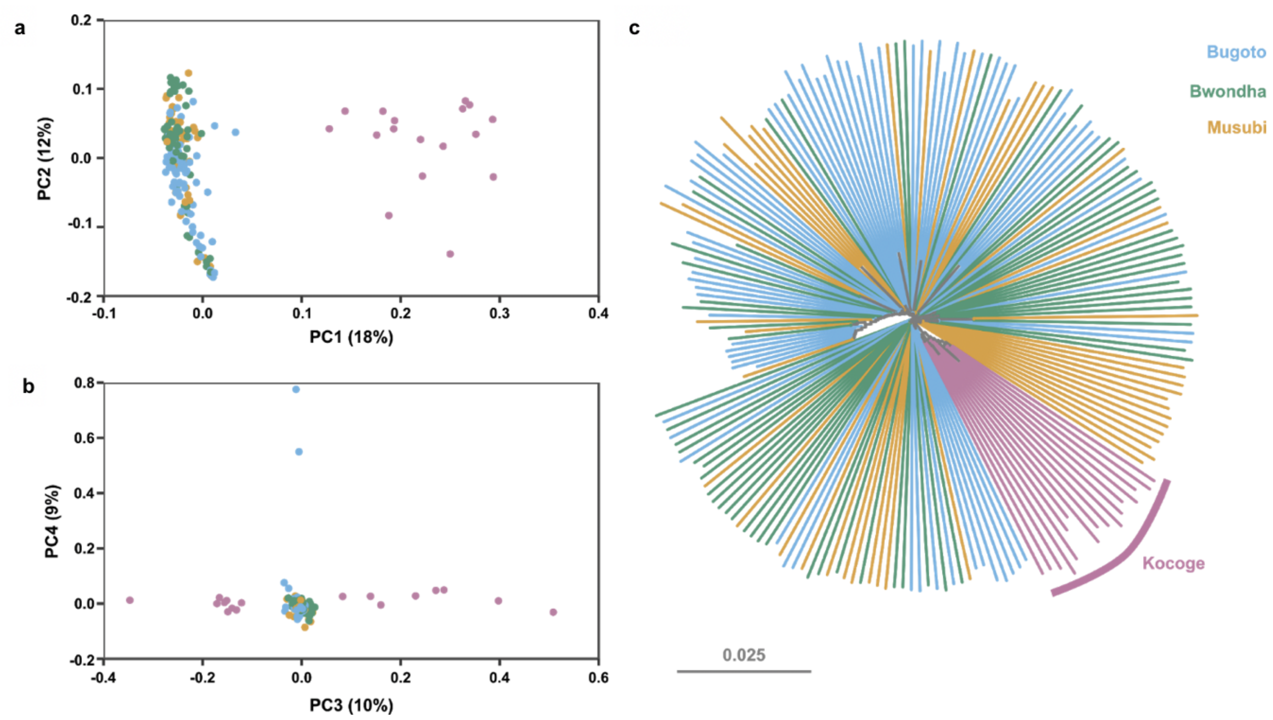

Supplementary Figure 3: *Schistosoma mansoni* population structure based on 198 study samples from Uganda. (a&b) Principal component analysis (PCA) of genetic differentiation within and between isolates, with the first four principal components accounting for 49% of the total variance. Bugoto (light blue), Bwonda (green) and Musubi (yellow) represent schools from Mayuge district and Kocoge (pink) is found within Tororo district. (b) Mid-point rooted neighbour-joining phylogeny illustrating relatedness between isolates, branches are colored based on the school where sample collection occurred.

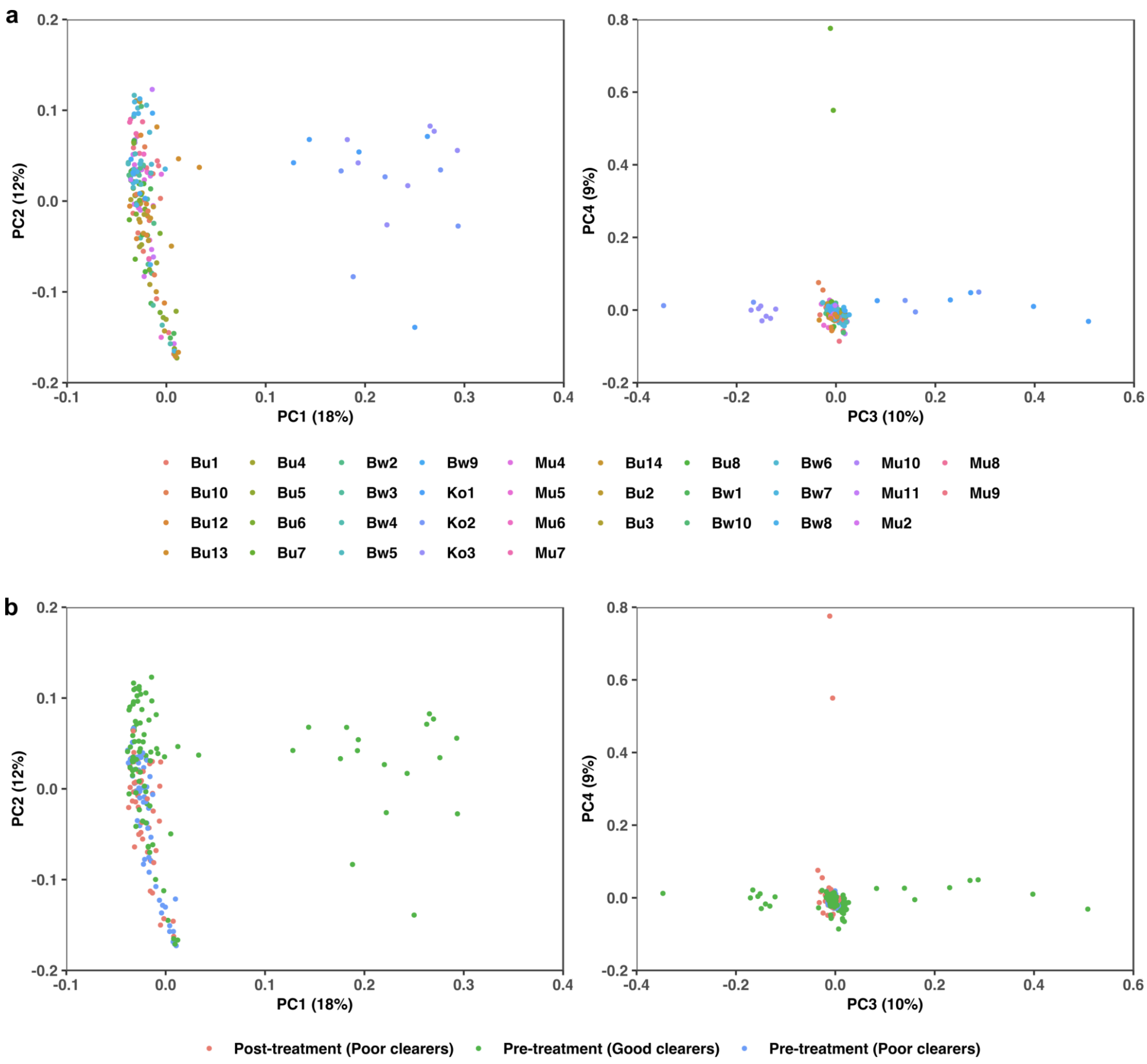

Supplementary Figure 4: Population structure in *S. mansoni* samples from Uganda overlaid with host or drug treatment-phenotype details. Principal component analysis (PCA) of genetic differentiation within and between 198 miracidia *S. mansoni* isolates. (a) Colours represent the 34 children from which miracidia were sampled. (b) Colours represent the two sampling time points, pre-treatment (if sampled prior to treatment) and post-treatment (if sampled 4 weeks post-treatment), children were defined as 'good clearers' if no sampled miracidia hatched from stool samples after treatment or 'poor clearers' if miracidia could be hatched following treatment.

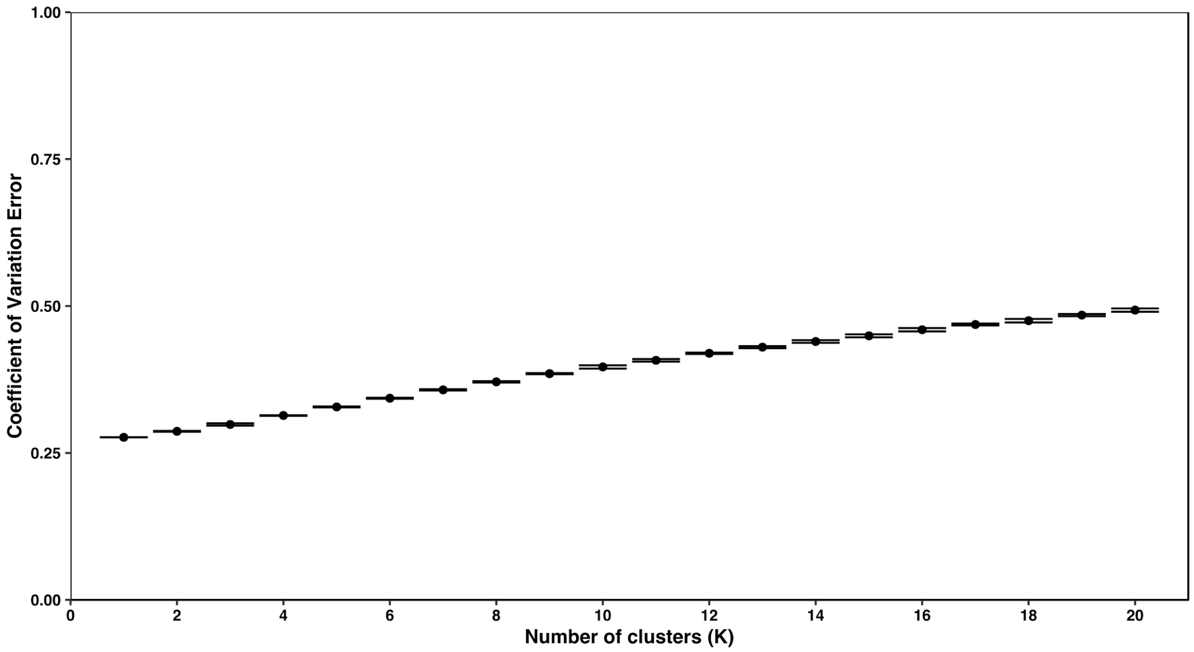

Supplementary Figure 5: Coefficient of variation (CV) values generated by ADMIXTURE with K values ranging from 1-20, 10-fold cross-validation and standard error estimation with 250 bootstraps. The analysis was repeated 10 times with different random seeds. Median CV scores and standard deviations are shown for each value of K.

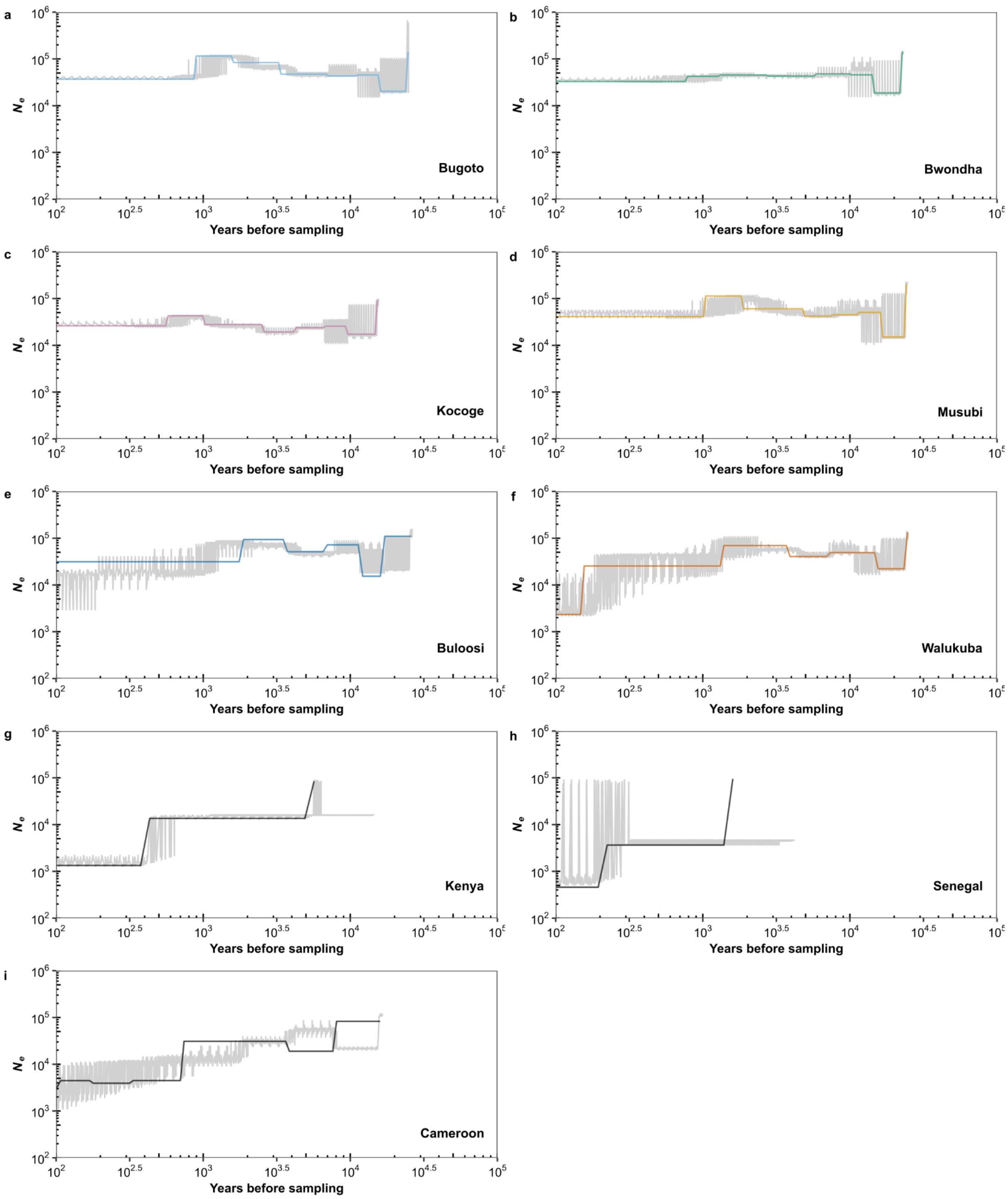

Supplementary Figure 6: Uncertainty in inference of demographic history using SMC++ for *S. mansoni* populations. SMC++ was run on each autosome using a per-generation mutation rate of 8.1e-9 and a generation time of 71 days. Bugoto (*n* = 75), Bwonda (*n* = 60), Musubi (*n* = 46) school populations were sampled from schools from Mayuge district (Uganda). Kocoge population (*n* = 17) were sampled from a school within Tororo district (Uganda). Walukuba population (*n* =1) was sampled from a school on the shoreline of Lake Albert (Uganda) and the Buloosi population (*n* = 1) was sampled on the shoreline of Lake Victoria approximately 40 km east of Mayuge district. Non-Ugandan subpopulations are from Senegal (*n* =1), Cameron (*n* =1) and Kenya (*n* =1). All samples from each population (for each school or for outgroups each country) were included as single populations. Individual plots show replicates used for each sample randomly subsampling 7.5 Mb from each chromosome 25 times for each population (grey lines) and colored lines show the original non-subsampled estimates from Supplementary Figure 7.

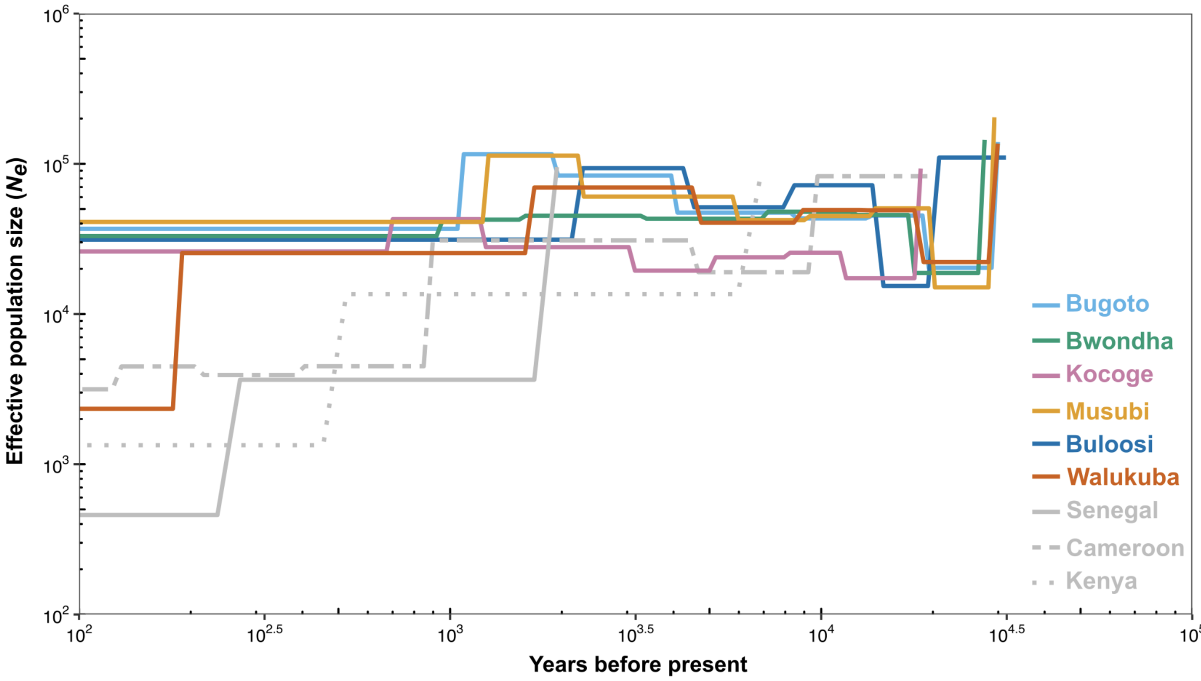

Supplementary Figure 7: Inference of demographic history using SMC++ for *S. mansoni* subpopulations. SMC++ was run on each autosome using a per-generation mutation rate of 8.1e-9 and a generation time of 71 days. Bugoto (n = 75), Bwonda (n = 60), Musubi (n = 46) school populations were sampled from schools from Mayuge district (Uganda). Kocoge population (n = 17) were sampled from a school within Tororo district (Uganda). Walukuba population (n =1) was sampled from a school on the shoreline of Lake Albert (Uganda) and the Buloosi population (n = 1) was sampled on the shoreline of Lake Victoria approximately 40 km east of Mayuge district. Non-Ugandan subpopulations are from Senegal (n =1), Cameron (n =1) and Kenya (n =1). All samples from each population (for each school or for outgroups each country) were included as single populations.

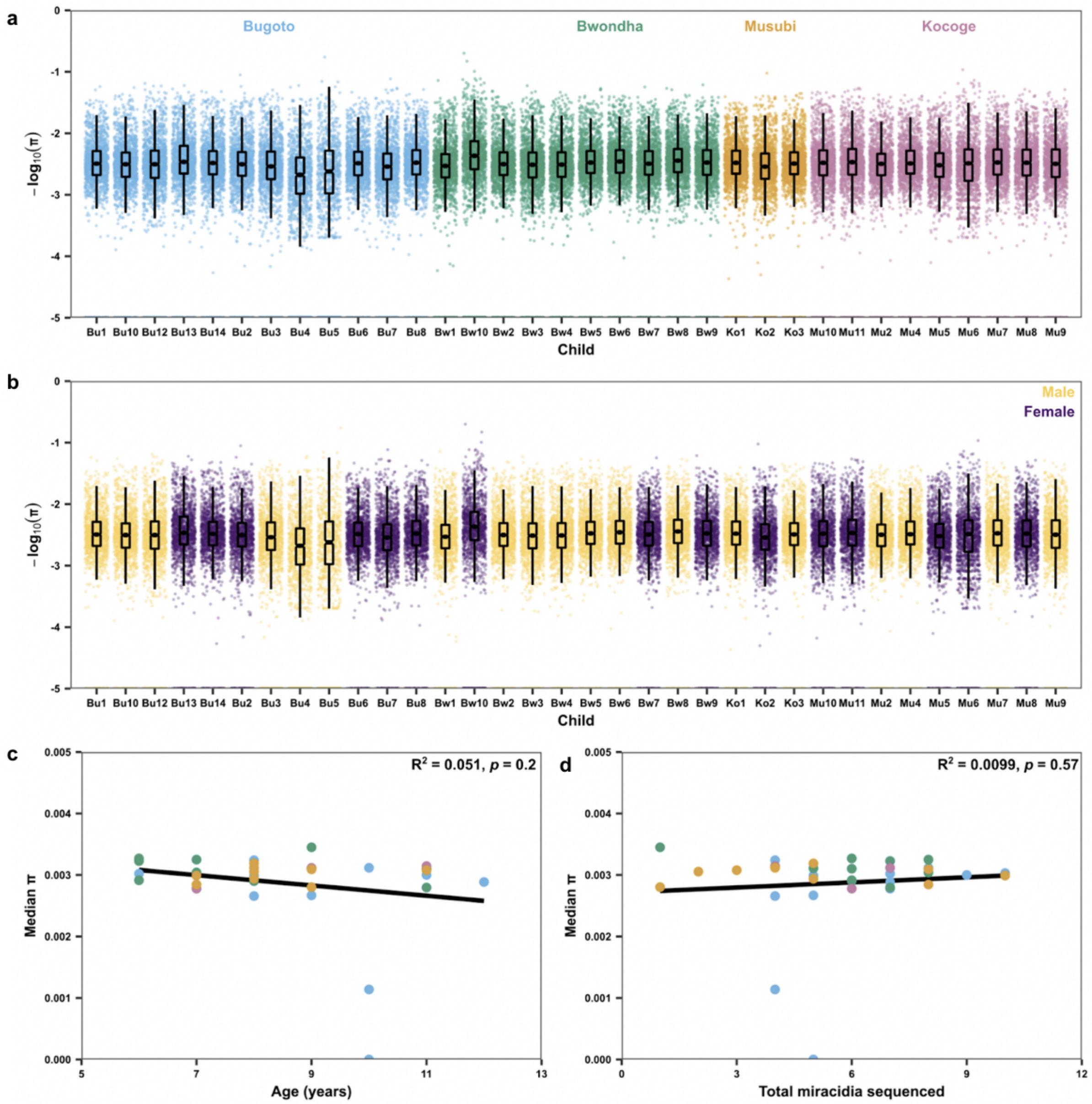

Supplementary Figure 8: Diversity of infrapopulations. (a) Nucleotide diversity (π) for each child's parasite infrapopulation. Nucleotide diversity was calculated in 5 kb non-overlapping windows along each autosome, each point represents a single window. X-axis values represent each child's infrapopulation, points are coloured by the school each child was attending at time of sampling. Individual infrapopulation sizes (number of miracidia sequenced from each infrapopulation): Bu1 = 10, Bu10 = 5, Bu12 = 7, Bu13 = 4, Bu14 = 8, Bu2 = 7, Bu3 = 5, Bu4 = 4, Bu5 = 5, Bu6 = 9, Bu7 = 4, Bu8 = 7, Bw1 = 7, Bw10 = 1, Bw2 = 8, Bw3 = 6, Bw4 = 6, Bw5 = 8, Bw6 = 7, Bw7 = 6, Bw8 = 6, Bw9 = 5, Ko1 = 5, Ko2 = 5, Ko3 = 7, Mu10 = 2, Mu11 = 4, Mu2 = 10, Mu4 = 8, Mu5 = 8, Mu6 = 1, Mu7 = 5, Mu8 = 3, Mu9 = 5. Samples from children Bu4 & Bu5 were excluded from population comparisons and summary statistics due to consistently low coverage across all samples. (b) Autosomal nucleotide diversity values from (a) coloured by the sex of each child. For all boxplots, the central line indicates the median, the top and bottom edges of the box indicate the 25th and 75th percentiles, respectively. The maximum whisker lengths are specified as 1.5 times the interquartile range. Median autosomal nucleotide diversity calculated for each infrapopulation and plotted against the child's age (c) and total infrapopulation size (d) coloured by the school each child was attending at time of sampling. For (c) and (d) pearson correlation coefficients and two-sided tests for significance levels for this correlation were calculated using the ggpubr 'stat_cor' function, no adjustments were made for multiple comparisons.

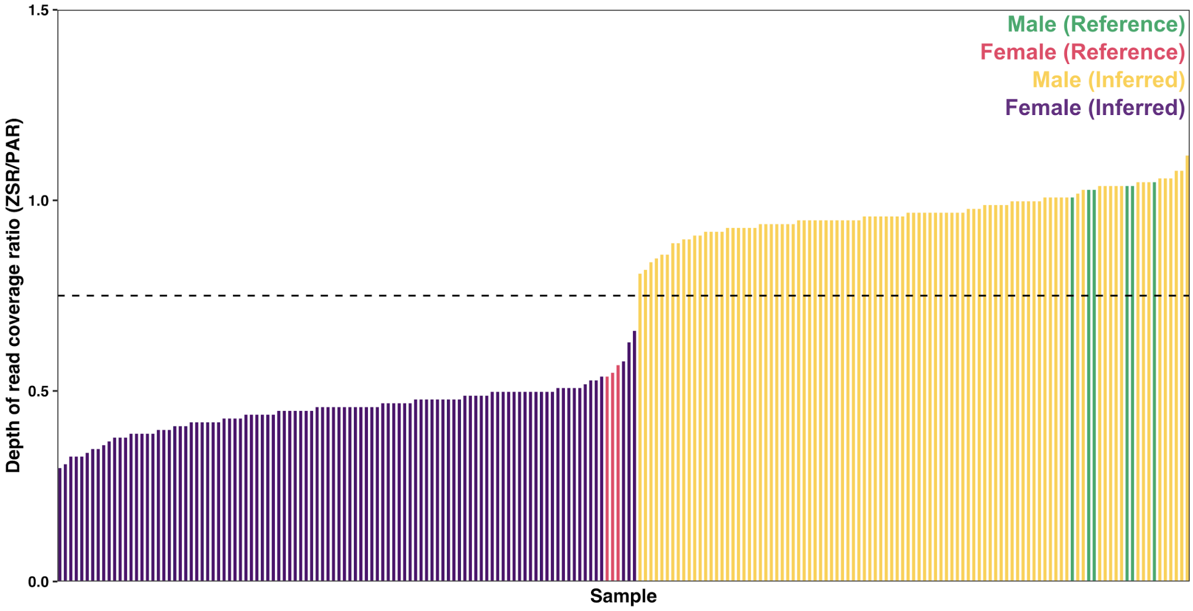

Supplementary Figure 9: Identification of sex of all samples using differential depth of coverage. For each sample the per-base read depth over the pseudoautosomal (PAR1 and PAR2) regions and the Z-specific region (ZSR) of the Z chromosome were calculated. Samples with a >0.75 ZSR/PAR ratio were designated as males. Samples with <0.75 ZSR/PAR ratio were designated females. Samples with known sex prior to sequencing are refered to as ‘reference’ samples, samples of unknown sex are referred to as having ‘inferred’ sex.

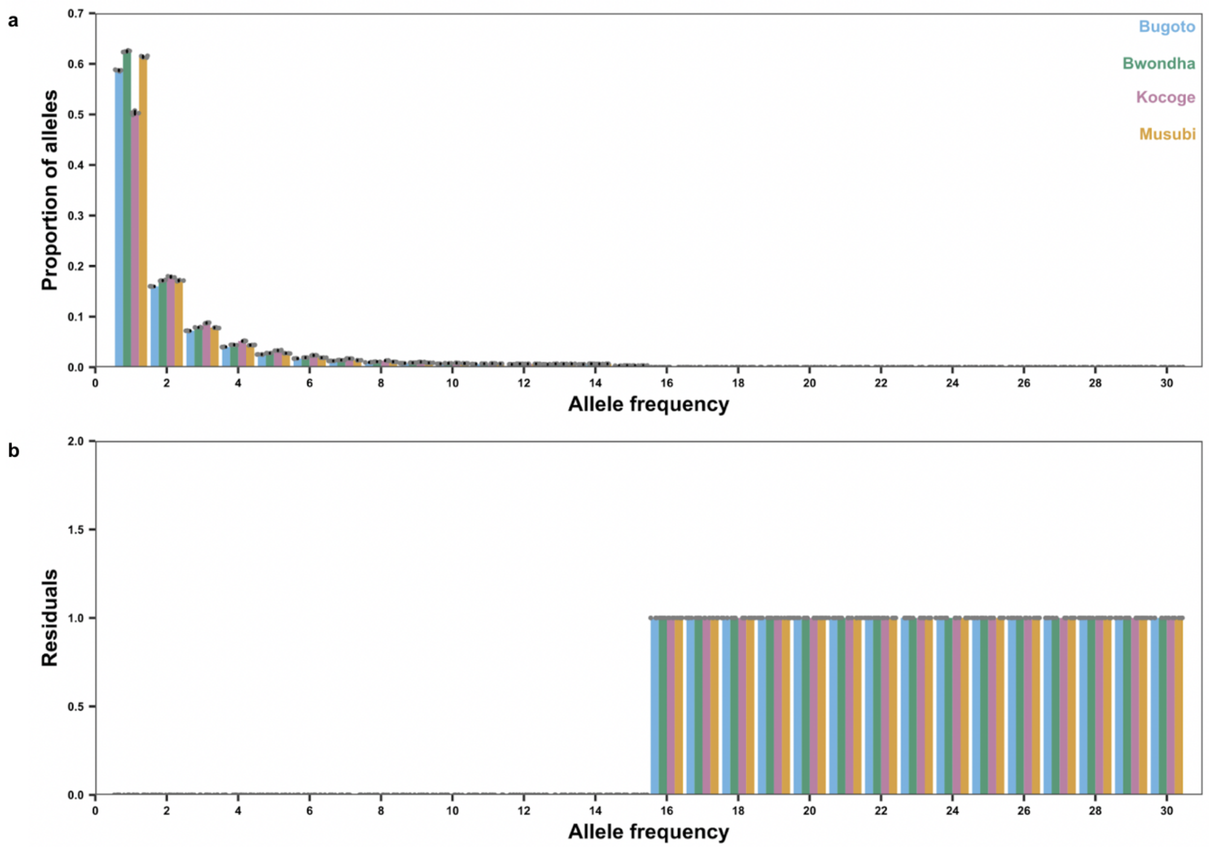

Supplementary Figure 10: Genome-wide allele frequency patterns. (a) One dimensional site frequency spectra for each parasite population sampled from children in each school: Bugoto (light blue), Bwondha (green), Musubi (yellow) and Kocoge (pink). For each school, miracidial populations were subsampled (*n* = 15 miracidia per school) and site frequency spectra were calculated, this was repeated for a total of five replicates. The x-axis represents the derived allele frequency and y-axis represents the proportion of sites at each allele frequency. Coloured bars represent the median proportion of sites across all replicates for each school, black error bars represent the standard deviation around the median for all replicates, grey points represent the individual results for each replicate. (b) Plot of residuals (normalised measure of the differences between inferred δaδi model and data) for the 1D-SFS. For both (a) and (b) bars represent the median proportion of sites across all replicates, error bars represent the standard deviation around the median for all replicates, grey points represent the individual results for each replicate.

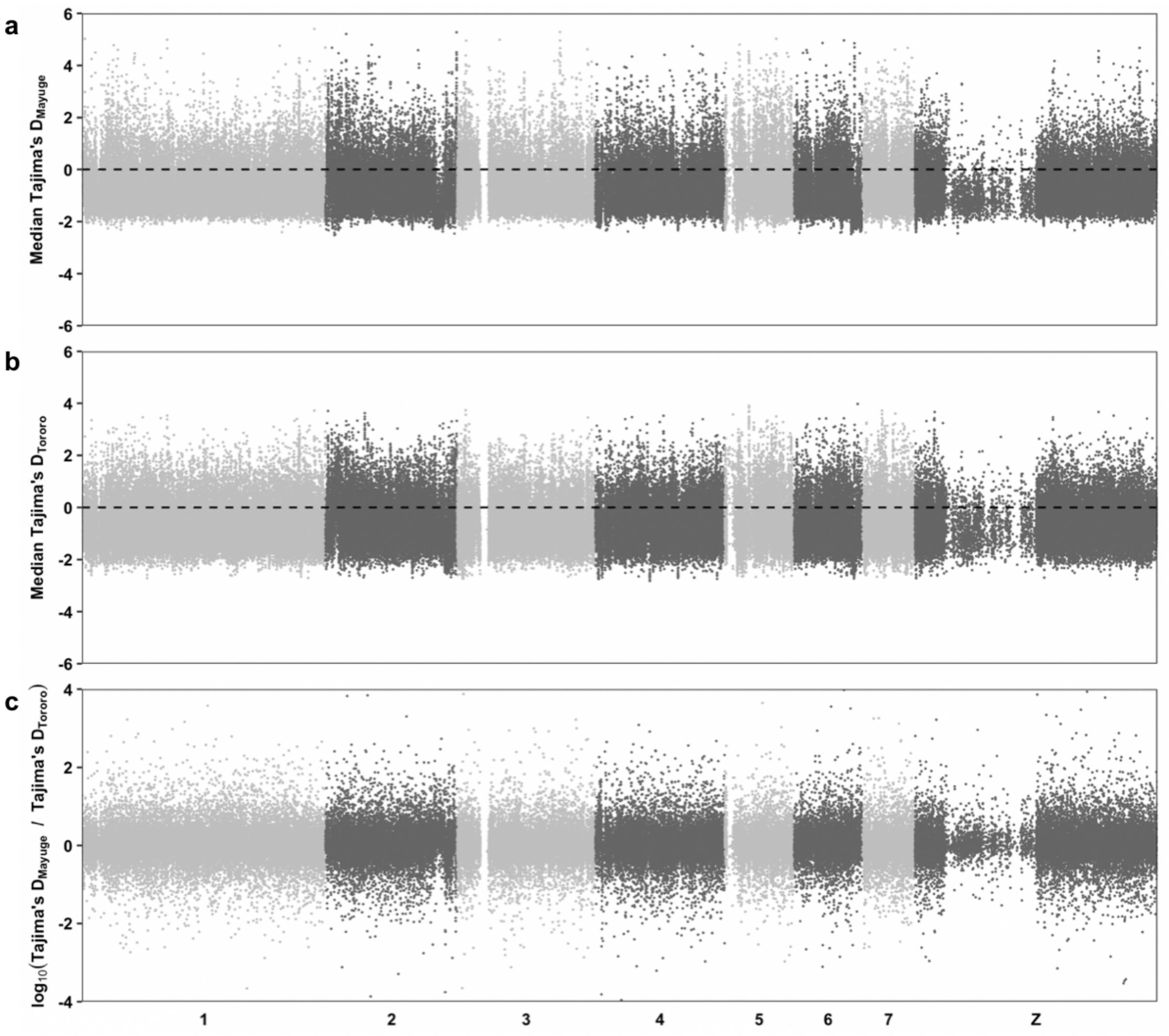

Supplementary Figure 11: Median Tajima's D values for each district population. Mean Tajima's D values were calculated in 2 kb non-overlapping windows along each chromosome (shown in alternating shades of grey) for each district population (a) Mayuge district (*n* = 181) and (b) Tororo district (*n* = 17). (c) The ratio of mean nucleotide diversity between the districts for each 2 kb window.

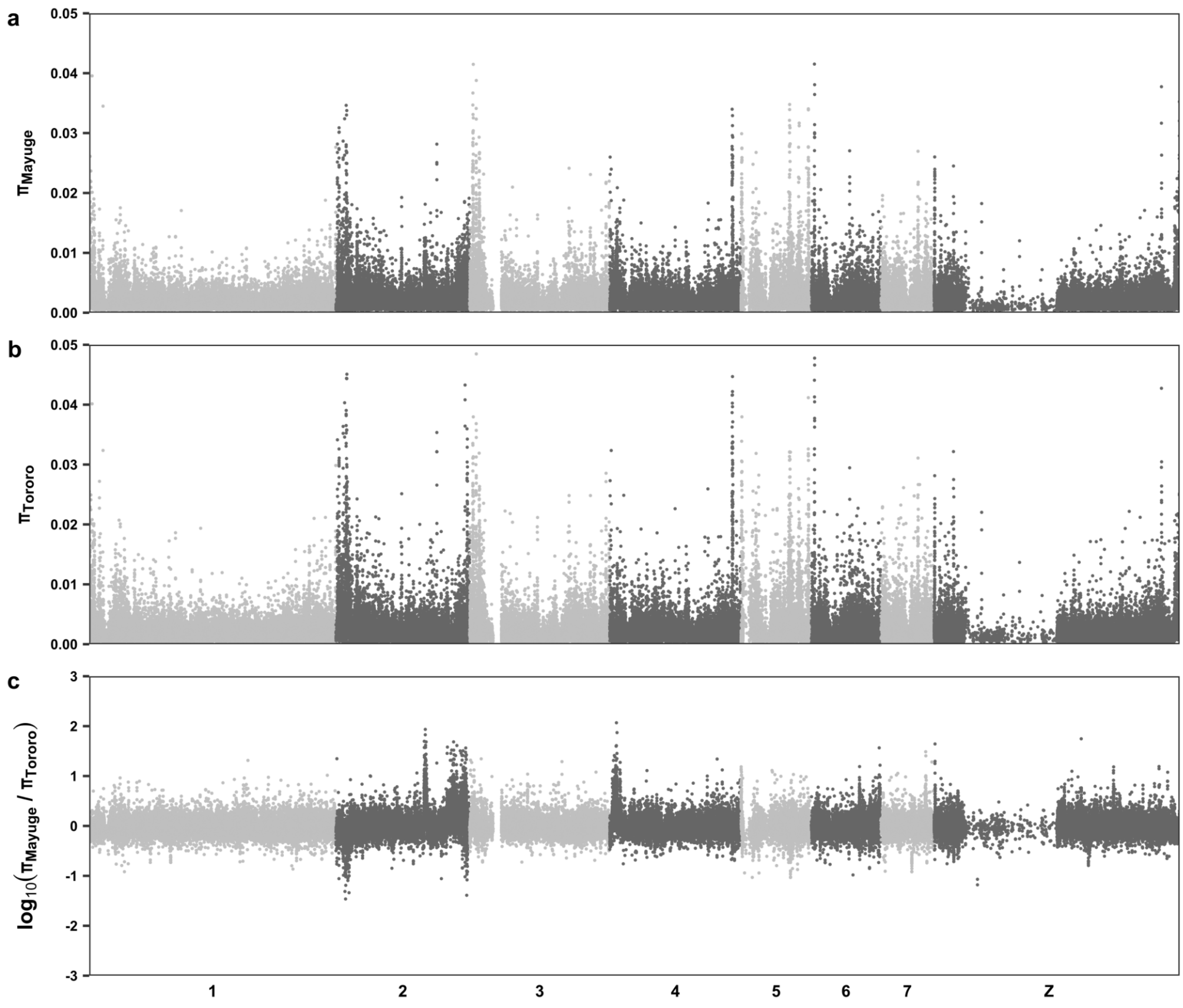

Supplementary Figure 12: Nucleotide diversity for each district population. Mean nucleotide diversity (π) was calculated in 2 kb non-overlapping windows along each chromosome (shown in alternating shades of grey) for each district population (a) Mayuge district (*n* = 181) and (b) Tororo district (*n* = 17). (c) The ratio of mean nucleotide diversity between the districts for each 2 kb window.

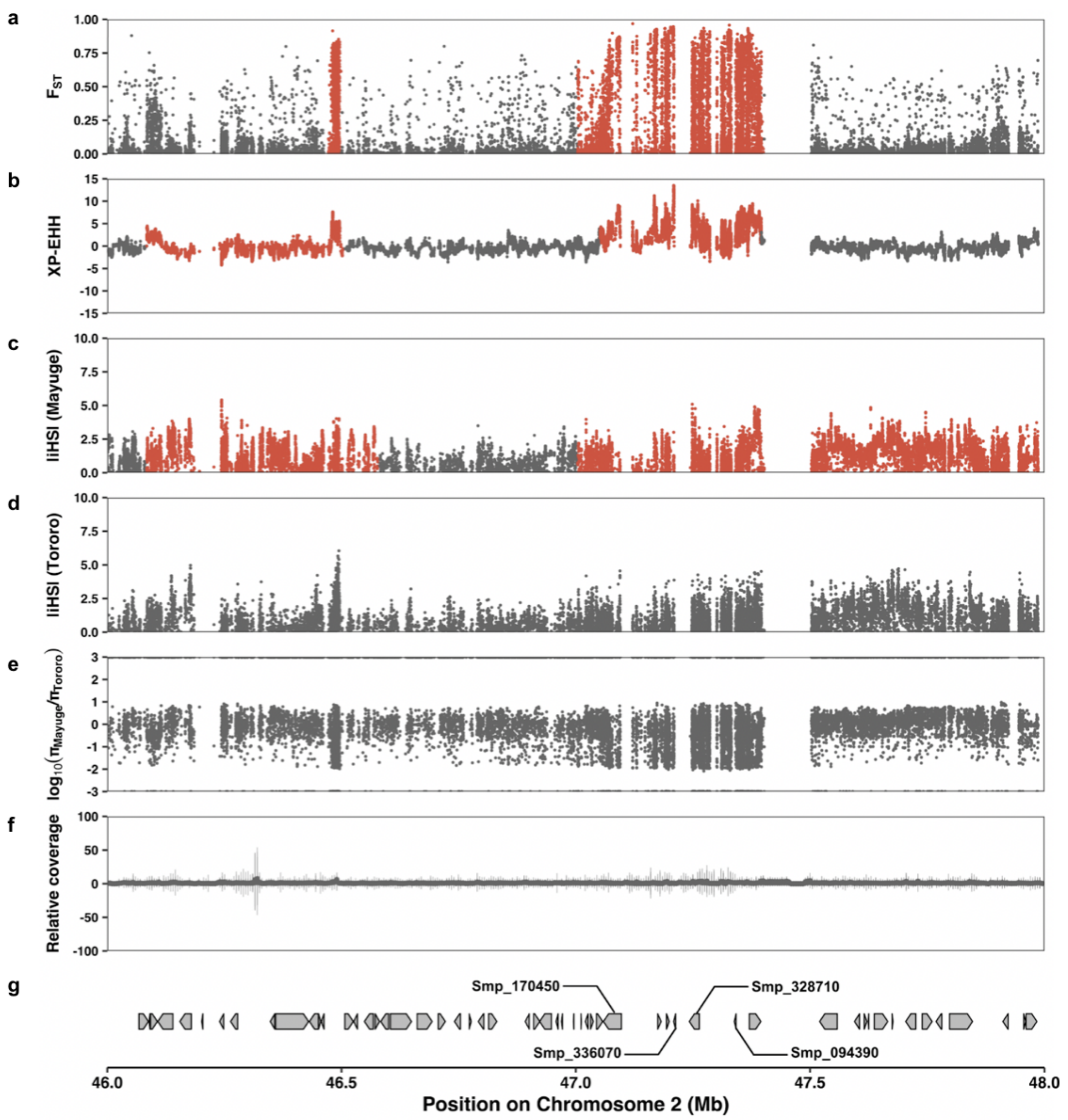

Supplementary Figure 13: Selection statistics over candidate regions of selection. Points in each window show the values of each metric of selection or supporting statistic for each biallelic single-nucleotide polymorphism (SNP) within a 2 Mb region of chromosome 2. (a) Fixation index (F_{ST}) and (b) cross-population extended haplotype homozygosity (XP-EHH) values were calculated between populations from Mayuge district (*n* = 181) a region of long-term MDA pressure (8-9 previous annual rounds), and Tororo district (*n* = 17), a region of short-term MDA pressure (one previous round with limited coverage). Integrated haplotype scores (iHS) for Mayuge district (c) and Tororo district (d). (e) The ratio of nucleotide diversity between each district calculated at each variant site. (f) Median depth of read coverage of 2 kb non-overlapping sliding windows, the median coverage was calculated for each sample and then this was divided by the median autosomal coverage for each sample, points represent the median coverage calculated across all 198 miracidial samples, vertical lines show the standard deviations of each window. (g) Location of genes within or adjacent to candidate regions of selection. Variants and windows in plots (a), (b) and (c) in red were found within candidate regions of selection (as indicated in Figure 4 and Supplementary Data 4) across one or more of the F_{ST}, XP-EHH or |iHS| (Mayuge only) statistics.

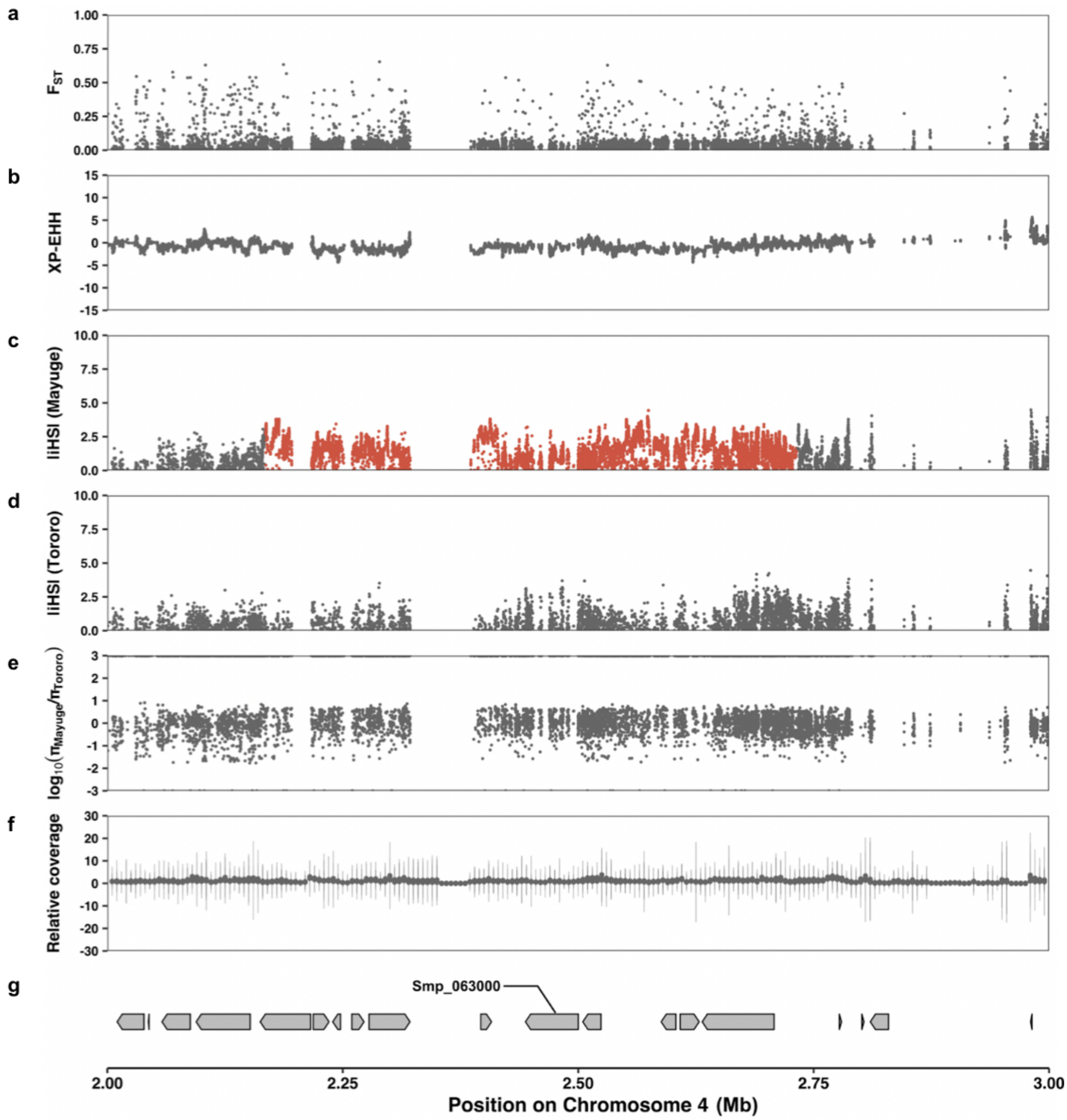

Supplementary Figure 14: Selection statistics over candidate regions of selection. Points in each window show the values of each metric of selection or supporting statistic for each biallelic single-nucleotide polymorphism (SNP) within a 1 Mb region of chromosome 4. (a) Fixation index (F_{ST}) and (b) cross-population extended haplotype homozygosity (XP-EHH) values were calculated between populations from Mayuge district (*n* = 181) a region of long-term MDA pressure (8-9 previous annual rounds), and Tororo district (*n* = 17), a region of short-term MDA pressure (one previous round with limited coverage). Integrated haplotype scores (iHS) for Mayuge district (c) and Tororo district (d). (e) The ratio of nucleotide diversity between each district calculated at each variant site. (f) Median depth of read coverage of 2 kb non-overlapping sliding windows, the median coverage was calculated for each sample and then this was divided by the median autosomal coverage for each sample, points represent the median coverage calculated across all 198 miracidial samples, vertical lines show the standard deviations of each window. (g) Location of genes within or adjacent to candidate regions of selection. Variants and windows in plots (a), (b) and (c) in red were found within candidate regions of selection (as indicated in Figure 4 and Supplementary Data 4) across one or more of the F_{ST}, XP-EHH or |iHS| (Mayuge only) statistics.

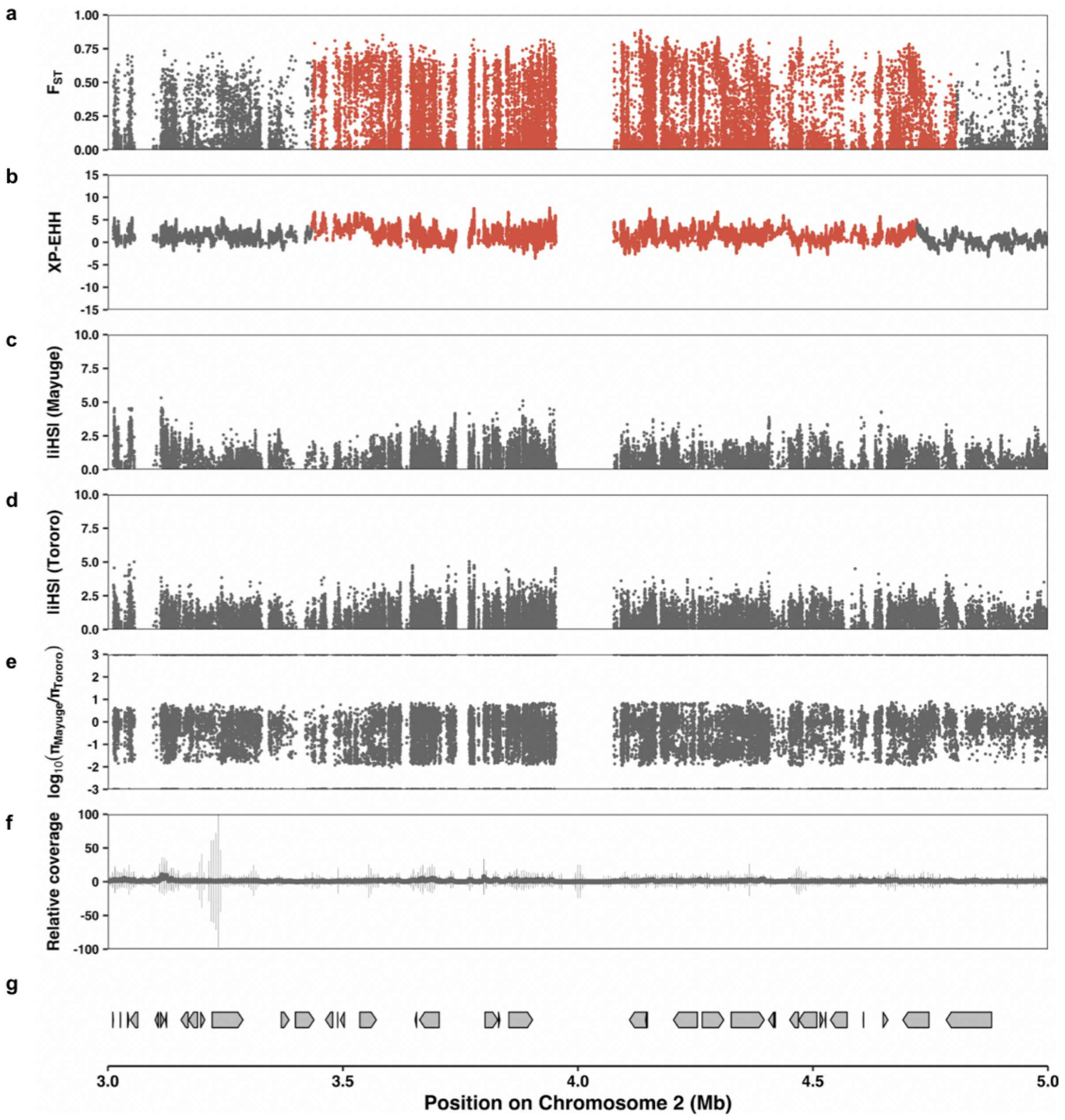

Supplementary Figure 15: Selection statistics over candidate regions of selection. Points in each window show the values of each metric of selection or supporting statistic for each biallelic single-nucleotide polymorphism (SNP) within a 2 Mb region of chromosome 2. (a) Fixation index (F_{ST}) and (b) cross-population extended haplotype homozygosity (XP-EHH) values were calculated between populations from Mayuge district (*n* = 181) a region of long-term MDA pressure (8-9 previous annual rounds), and Tororo district (*n* = 17), a region of short-term MDA pressure (one previous round with limited coverage). Integrated haplotype scores (iHS) for Mayuge district (c) and Tororo district (d). (e) The ratio of nucleotide diversity between each district calculated at each variant site. (f) Median depth of read coverage of 2 kb non-overlapping sliding windows, the median coverage was calculated for each sample and then this was divided by the median autosomal coverage for each sample, points represent the median coverage calculated across all 198 miracidial samples, vertical lines show the standard deviations of each window. (g) Location of genes within or adjacent to candidate regions of selection. Variants and windows in plots (a), (b) and (c) in red were found within candidate regions of selection (as indicated in Figure 4 and Supplementary Data 4) across one or more of the F_{ST}, XP-EHH or |iHS| (Mayuge only) statistics.

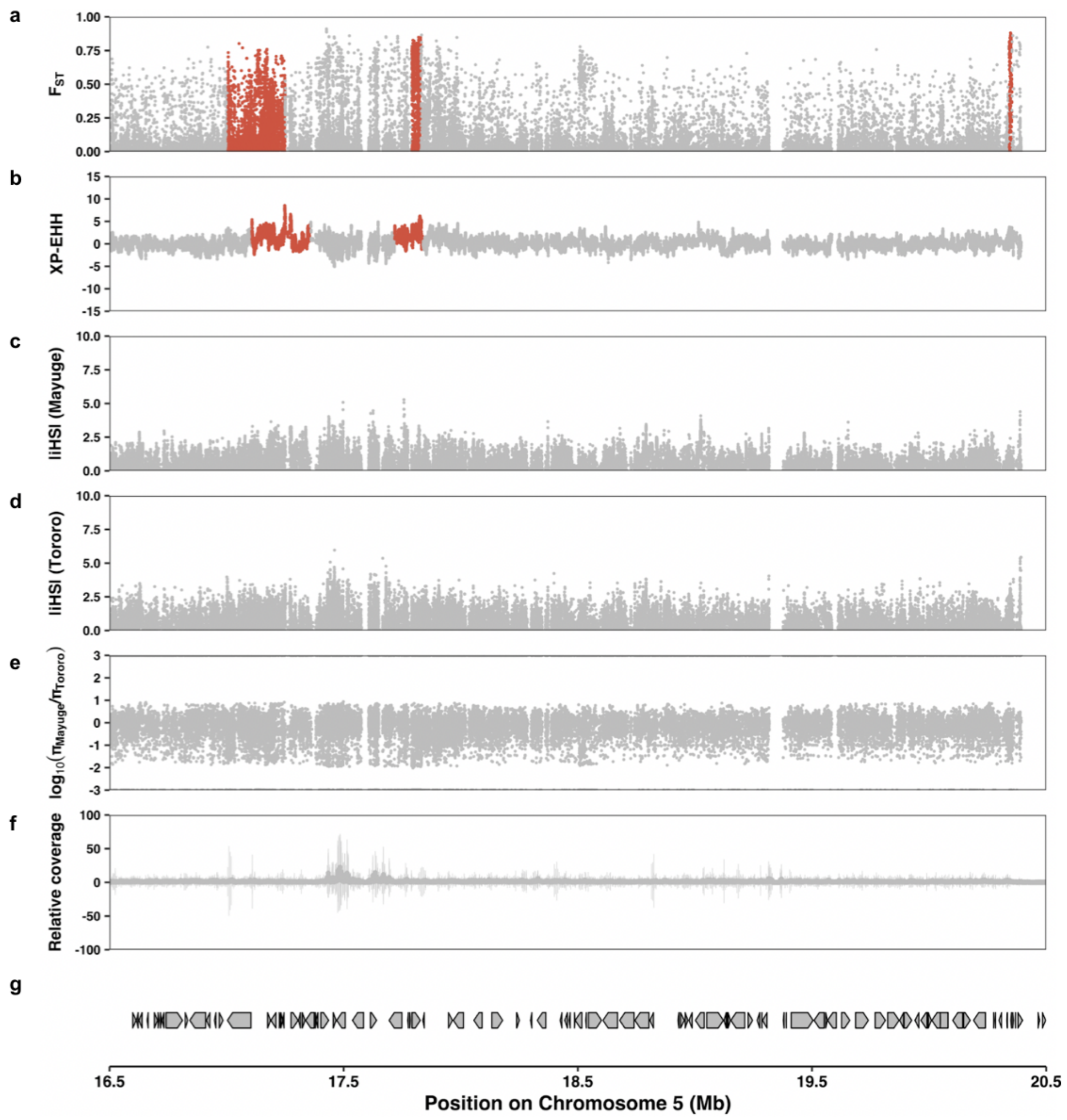

Supplementary Figure 16: Selection statistics over candidate regions of selection. Points in each window show the values of each metric of selection or supporting statistic for each biallelic single-nucleotide polymorphism (SNP) within a 4 Mb region of chromosome 5. (a) Fixation index (F_{ST}) and (b) cross-population extended haplotype homozygosity (XP-EHH) values were calculated between populations from Mayuge district (*n* = 181) a region of long-term MDA pressure (8-9 previous annual rounds), and Tororo district (*n* = 17), a region of short-term MDA pressure (one previous round with limited coverage). Integrated haplotype scores (iHS) for Mayuge district (c) and Tororo district (d). (e) The ratio of nucleotide diversity between each district calculated at each variant site. (f) Median depth of read coverage of 2 kb non-overlapping sliding windows, the median coverage was calculated for each sample and then this was divided by the median autosomal coverage for each sample, points represent the median coverage calculated across all 198 miracidial samples, vertical lines show the standard deviations of each window. (g) Location of genes within or adjacent to candidate regions of selection. Variants and windows in plots (a), (b) and (c) in red were found within candidate regions of selection (as indicated in Figure 4 and Supplementary Data 4) across one or more of the F_{ST}, XP-EHH or |iHS| (Mayuge only) statistics.

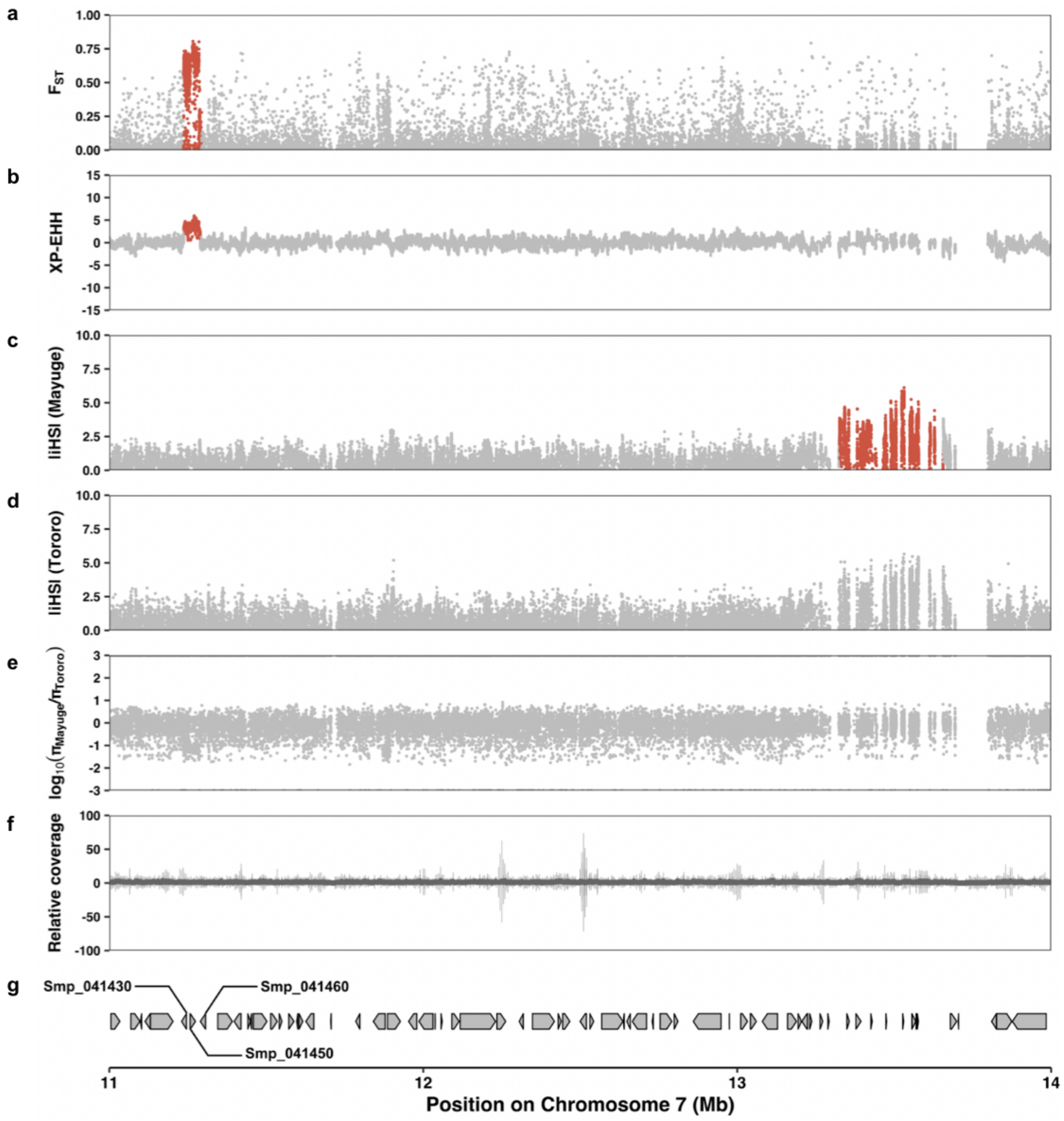

Supplementary Figure 17: Selection statistics over candidate regions of selection. Points in each window show the values of each metric of selection or supporting statistic for each biallelic single-nucleotide polymorphism (SNP) within a 3 Mb region of chromosome 7. (a) Fixation index (F_{ST}) and (b) cross-population extended haplotype homozygosity (XP-EHH) values were calculated between populations from Mayuge district (*n* = 181) a region of long-term MDA pressure (8-9 previous annual rounds), and Tororo district (*n* = 17), a region of short-term MDA pressure (one previous round with limited coverage). Integrated haplotype scores (iHS) for Mayuge district (c) and Tororo district (d). (e) The ratio of nucleotide diversity between each district calculated at each variant site. (f) Median depth of read coverage of 2 kb non-overlapping sliding windows, the median coverage was calculated for each sample and then this was divided by the median autosomal coverage for each sample, points represent the median coverage calculated across all 198 miracidial samples, vertical lines show the standard deviations of each window. (g) Location of genes within or adjacent to candidate regions of selection. Variants and windows in plots (a), (b) and (c) in red were found within candidate regions of selection (as indicated in Figure 4 and Supplementary Data 4) across one or more of the F_{ST}, XP-EHH or |iHS| (Mayuge only) statistics.

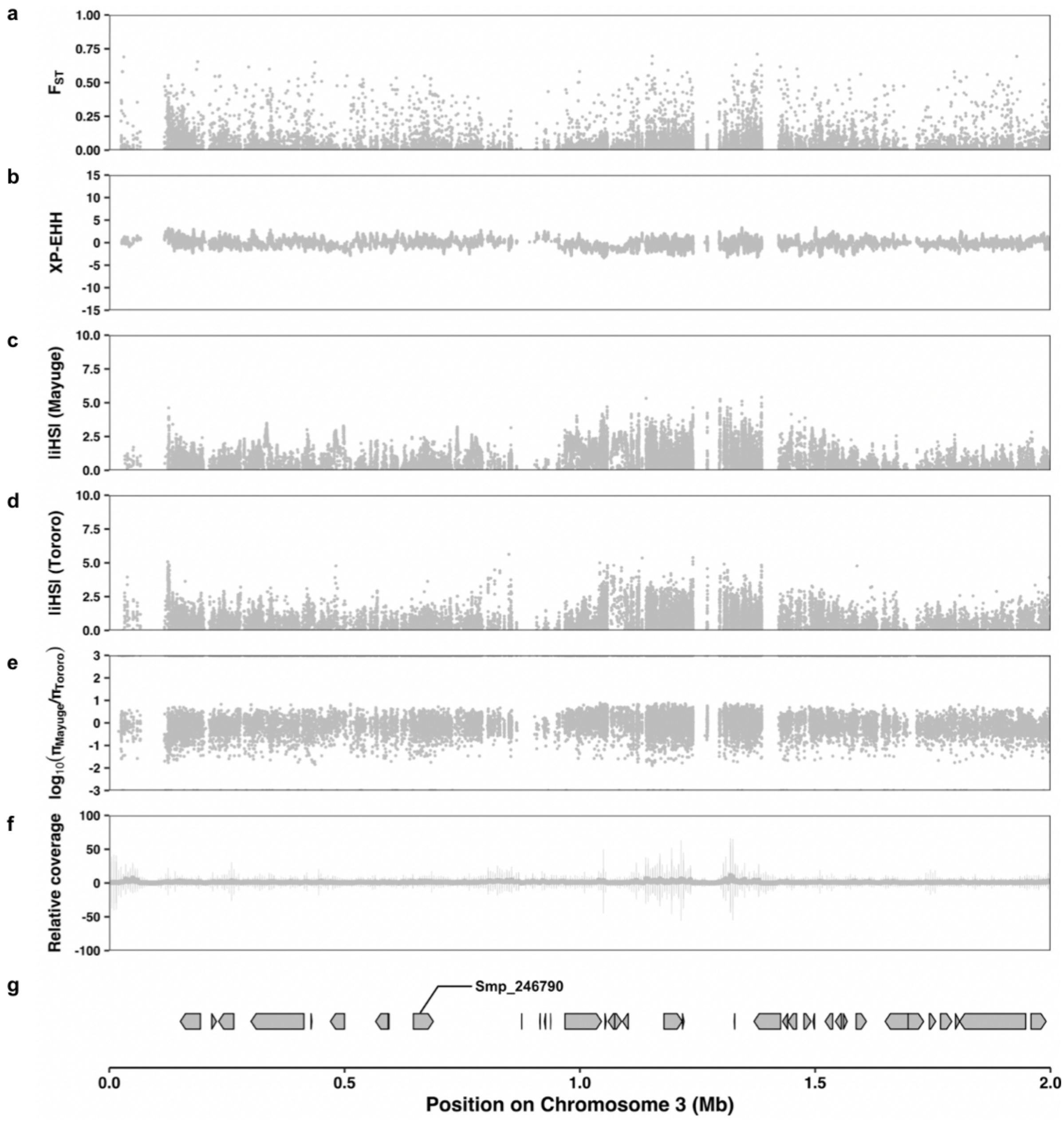

Supplementary Figure 18: Selection statistics over a non-candidate region. Points in each window show the values of each metric of selection or supporting statistic for each biallelic single-nucleotide polymorphism (SNP) within a 2 Mb region of chromosome 3. (a) Fixation index (F_{ST}) and (b) cross-population extended haplotype homozygosity (XP-EHH) values were calculated between populations from Mayuge district (*n* = 181) a region of long-term MDA pressure (8-9 previous annual rounds), and Tororo district (*n* = 17), a region of short-term MDA pressure (one previous round with limited coverage). Integrated haplotype scores (iHS) for Mayuge district (c) and Tororo district (d). (e) The ratio of nucleotide diversity between each district calculated at each variant site. (f) Median depth of read coverage of 2 kb non-overlapping sliding windows, the median coverage was calculated for each sample and then this was divided by the median autosomal coverage for each sample, points represent the median coverage calculated across all 198 miracidial samples, vertical lines show the standard deviations of each window. (g) Location of genes within or adjacent to candidate regions of selection. Variants and windows in plots (a), (b) and (c) in red were found within candidate regions of selection (as indicated in Figure 4 and Supplementary Data 4) across one or more of the F_{ST}, XP-EHH or |iHS| (Mayuge only) statistics.

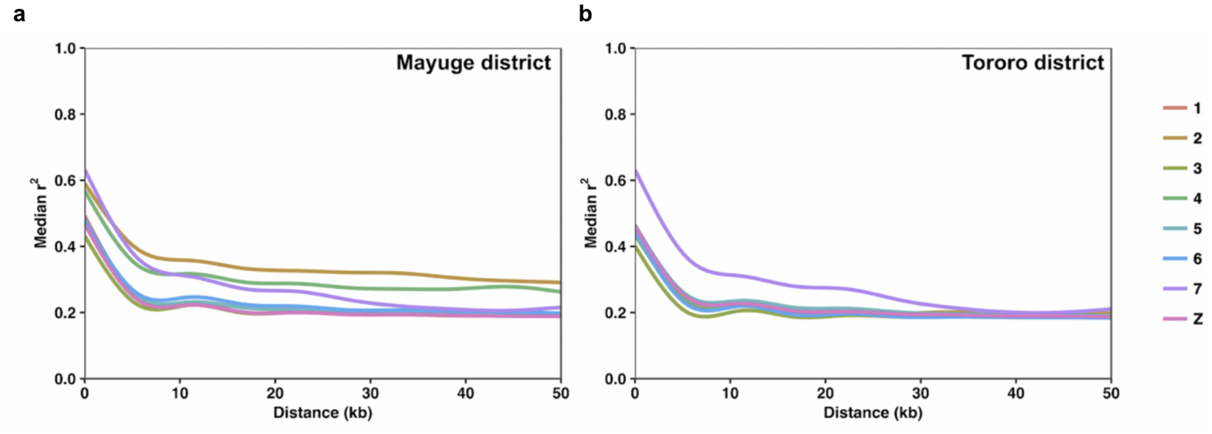

Supplementary Figure 19: The decay of linkage disequilibrium as the squared correlation of allele frequency (r^2) against distance (bp). Linkage was calculated between single-nucleotide polymorphisms within 50 kb per chromosome (1-Z) for each population separately. (a) Mayuge district school (Bugoto, Bwondha and Musubi schools) population (n = 181) was randomly subsampled to 17 miracidia, using five independent groups of samples and the median across all replicates was taken. (b) fTororo district (Kocoge school) population (n = 17), no subsampling was done. For each chromosome and district population the smoothed conditional mean is shown.
