# Supplementary material for: Whole-genome sequencing of Schistosoma mansoni reveals extensive diversity with limited selection despite mass drug administration
Source: Nat Commun. 2021 Aug 6;12:4776. doi: 10.1038/s41467-021-24958-0 (PMC8346512; doi:10.1038/s41467-021-24958-0)
Supplement: Supplementary file 3 — Description of Additional Supplementary Files [file 41467_2021_24958_MOESM3_ESM.pdf]

DESCRIPTIONS OF ADDITIONAL SUPPLEMENTARY FILES

File name: Supplementary Data 1
Title: Depth of read coverage statistics.
Description: Columns show total number of reads, the proportion of reads mapping to the *Schistosoma mansoni* genome and the proportion of reads mapped with mapping quality (MQ) values > 40.

File name: Supplementary Data 2
Title: Comparison of host infrapopulations.
Description: Fixation index (F_{ST}) and absolute divergence (d_{XY}), were calculated in 5 kb sliding, non-overlapping windows across each autosome calculated using PIXY (v.0.95.01). Population 1 & 2 refer to the miracidial infrapopulations sampled from each child.

File name: Supplementary Data 3
Title: Determination of miracidial sex.
Description: Median read depth over the pseudoautosomal regions (PAR1 and PAR2) and Z-specific regions (ZSR) of the Z chromosome. The ration between PAR and ZSR regions was used to infer the sex of miracidia. A ratio <0.75 indicates female miracidia, >0.75 indicates a male.

File name: Supplementary Data 4
Title: Genomic regions with evidence of positive selection.
Description: Described are regions of continuous elevated genome-wide integrated haplotype scores (IHS) scores within the Mayuge population (n = 181). In addition, regions of elevated genome-wide cross-population extended haplotype heterozygosity (XP-EHH) and genetic differentiation (F_{ST}) values between populations from Mayuge district (n = 181) and Tororo district (n = 17) are indicated. Median values for all variants were calculated in 2 kb non-overlapping windows along the eight *Schistosoma mansoni* chromosomes. Regions of consistent extreme values in either test were taken as putative regions under selection and windows within 300 kb were joined to form selected regions before being manually inspected and refined to encompass peak structures.

File name: Supplementary Data 5
Title: Non-redundant genomic regions with evidence of positive selection.
Description: Regions from Supplementary Data 5 were collapsed into non-redundant genomic regions with evidence of positive selection (identified using iHS, FST and XP-EHH scores).

File name: Supplementary Data 6
Title: Genes found within regions of positive selection.
Description: List of *Schistosoma mansoni* genes found within genomic regions under positive selection identified in Supplementary Data 5.

File name: Supplementary Data 7
Title: Variants within genes found in regions of positive selection.
Description: List of variants found within genes in regions under positive selection (Supplementary data 6) and impact of each variant as determined by SnpEff (intergenic variants were excluded).

File name: Supplementary Data 8
Title: Association between egg reduction rate and genomic variants.

Description: Variants found to be significantly associated with reduced egg reduction rate (ERR) estimates. A linear regression genome-wide association study was conducted using PLINK (v1.9) using the ERR estimates across all 181 Mayuge samples and 281,954 autosomal variants not found to be in strong linkage disequilibrium. The first four principal components of the PCA (figure 4) were used as covariates. A Bonferroni corrected significance threshold was used to determine a significance cutoff. For each variant the SnpEff predicted impact is also shown.

File name: Supplementary Data 9
Title: Summary statistics for each child.
Description: Pre- and post-treatment miracidia refer to miracidia sampled prior to treatment with praziquantel and four weeks post praziquantel treatment.

File name: Supplementary Data 10
Title: Read sequence metadata.
Description: Raw illumina reads used for population genomic analyses, location of reads and associated metadata.

File name: Supplementary Data 11
Title: *Schistosoma mansoni* (v.7) reference genome chromosome and scaffold classifications.
Description: Retained means the scaffolds or contigs were included in the read mapping, excluded means the scaffolds or contigs were excluded from read mapping.

File name: Supplementary Data 12
Title: Read contamination.
Description: Summary of proportion of sequence data classified as contaminants by Kraken.

File name: Supplementary Data 13
Title: Variant summary statistics.
Description: Per sample summary statistics of post-quality control variant calls as classified by BCFtools.
File name: Supplementary Data 14
Title: Variants with eleveated fixation index scores.
Description: Per variant site fixation index (FST) values across all chromosomes (only showing FST>=0.9).
