# Supplementary material for: Whole-genome sequencing of Schistosoma mansoni reveals extensive diversity with limited selection despite mass drug administration
Source: Nat Commun. 2021 Aug 6;12:4776. doi: 10.1038/s41467-021-24958-0 (PMC8346512; doi:10.1038/s41467-021-24958-0)
Supplement: Supplementary file 18 — Reporting Summary [file 41467_2021_24958_MOESM18_ESM.pdf]

nature research

Corresponding author(s): Duncan Berger, Joanne Webster, James Cotton, Matthew Berriman

Last updated by author(s): Aug 26, 2020

Reporting Summary

Nature Research wishes to improve the reproducibility of the work that we publish. This form provides structure for consistency and transparency in reporting. For further information on Nature Research policies, see our Editorial Policies and the Editorial Policy Checklist.

Statistics

For all statistical analyses, confirm that the following items are present in the figure legend, table legend, main text, or Methods section.

n/a	Confirmed	
☐	☒	The exact sample size (n) for each experimental group/condition, given as a discrete number and unit of measurement
☐	☒	A statement on whether measurements were taken from distinct samples or whether the same sample was measured repeatedly
☐	☒	The statistical test(s) used AND whether they are one- or two-sided<br>*Only common tests should be described solely by name; describe more complex techniques in the Methods section.*
☐	☒	A description of all covariates tested
☐	☒	A description of any assumptions or corrections, such as tests of normality and adjustment for multiple comparisons
☐	☒	A full description of the statistical parameters including central tendency (e.g. means) or other basic estimates (e.g. regression coefficient) AND variation (e.g. standard deviation) or associated estimates of uncertainty (e.g. confidence intervals)
☐	☒	For null hypothesis testing, the test statistic (e.g. F, t, r) with confidence intervals, effect sizes, degrees of freedom and P value noted<br>*Give P values as exact values whenever suitable.*
☒	☐	For Bayesian analysis, information on the choice of priors and Markov chain Monte Carlo settings
☒	☐	For hierarchical and complex designs, identification of the appropriate level for tests and full reporting of outcomes
☒	☐	Estimates of effect sizes (e.g. Cohen's d, Pearson's r), indicating how they were calculated

Our web collection on statistics for biologists contains articles on many of the points above.

Software and code

Policy information about availability of computer code

Data collection	No software was used for data collection.
Data analysis	Custom code and workflows can be found here: https://github.com/duncanberger/PZQ_POPGEN, [https://doi.org/10.5281/zenodo.5013733]<br><br>Open-source software used: Kraken (v.0.10.6), BWA mem (v.0.7.17) , GATK (v.4.1.0.0), PicardTools MarkDuplicates (as part of GATK v.4.1.0.0), VCFtools (v.0.1.15), KING (v.2.1.5), PLINK (v.2.0), PLINK (v.1.9), PIXY (v.0.95.01), Beagle (v.5.0), Selscan (v.1.2.0a), QGIS (v.3.2.2), GNU datamash (v1.4), R (3.5.1), bcftools (v.1.9), GNU parallel (v.20180122), seqtk (v.1.3 (r106)), ADMIXTURE (v.1.3), Bedtools (v2.30.0), snpEff (v.4.3t), norm (v.1.2.0a), SMC++ (V1.15.2), easySFS (https://github.com/isaacovercast/easySFS), δaδi (v.2.7.0).<br><br>R packages: phangorn (v.2.4.0), ggtree (v.1.10.5), ggplot2 (v.3.1.0), cowplot (v.0.9.3), ape (v.5.2), MCMCglmm (v.2.32).

For manuscripts utilizing custom algorithms or software that are central to the research but not yet described in published literature, software must be made available to editors and reviewers. We strongly encourage code deposition in a community repository (e.g. GitHub). See the Nature Research guidelines for submitting code & software for further information.

Data

Policy information about availability of data

All manuscripts must include a data availability statement. This statement should provide the following information, where applicable:

- Accession codes, unique identifiers, or web links for publicly available datasets
- A list of figures that have associated raw data
- A description of any restrictions on data availability

Data availability
The sequencing data generated in this study have been deposited in the European Nucleotide Archive (ENA) repository under accession code PRJEB31375 [https://www.ebi.ac.uk/ena/browser/view/PRJEB31375]. Individual sample accessions are listed in Supplementary Data 10. Source data are available at [http://doi.org/10.5281/zenodo.4940588]. Genome and annotation files are available through WormBase Parasite [https://parasite.wormbase.org/Schistosoma_mansoni_prjea36577/Info/Index/]. Egg count data used to produce the egg reduction rate estimates are available [10.13140/RG.2.2.12687.84640]. To reduce the number of indirect identifiers available children's ages have been removed, access can be obtained upon reasonable request to the corresponding authors.

Field-specific reporting

Please select the one below that is the best fit for your research. If you are not sure, read the appropriate sections before making your selection.

☐ Life sciences ☐ Behavioural & social sciences ☒ Ecological, evolutionary & environmental sciences

For a reference copy of the document with all sections, see nature.com/documents/nr-reporting-summary-flat.pdf

Ecological, evolutionary & environmental sciences study design

All studies must disclose on these points even when the disclosure is negative.

Study description	A cross-sectional survey conducted in 2014 by Crellen et al. (2016) examining children infected with Schistosoma mansoni attending schools in Uganda found a statistically significant drop in praziquantel efficacy in schools that had been part of long-term mass-drug administration (MDA) programmes. As part of that survey miracidia (larvae) were collected from 414 children (aged 6-12 years) before and (where applicable) 25-27 days after treatment. Our manuscript describes the whole-genome sequencing and analysis of 198 miracidia (222 before variant calling quality control) collected from 34 of the 414 children sampled during that survey.<br><br>Sample collection, classification and ethical approval have been described previously by Crellen et al. 2016 but for clarity we have included details on the sampling strategies, inclusion criteria and ethical approval in this reporting summary.
Research sample	Miracidia (larvae) of Schistosoma mansoni isolated on Whatman FTA cards (one miracidia per spot) derived from stool samples. Originally sampled during a survey by Crellen et al. (2016).
Sampling strategy	Total sample size were based on sequencing cost limitations (the amount of sequence data that could be generated for size of the Schistosoma mansoni genome) as well as the amount of available parasite material from each site.<br><br>Within schools populations we aimed to sequence five randomly selected miracidia per child before treatment and, in the case of poor clearing individuals, five randomly selected miracidia after treatment. In practice there was not always sufficient parasite material, particularly from post-treatment egg collections, and so the number of miracidia at each time-point varied between 1-9. In total we selected 222 miracidia, of which 204 were from 31 children in schools in Mayuge district (Bugoto n = 86, Bwondha n = 67, Musubi n = 51) and 18 were from 3 children Kocoge school, in Tororo district. Overall 165 miracidia were sampled before treatment with praziquantel and 57 miracidia were from stool samples collected after treatment. A smaller number of samples were sequenced from the Tororo district due to a limited amount of available samples from the Kocoge school.
Data collection	TC, MB, JPW and JAC conceived of the project, for which MB and JPW secured funding. TC, NBK, EMT, PHL and JPW planned and coordinated the fieldwork, which was carried out by TC, PHL and MA. Information on the children was recorded using a pen and paper at recruitment. Egg counts were also recorded using pen and paper. Miracidia were isolated from a petri dish of filtered stool using a pipette and dissecting microscope as described in the Methods. Details on the isolated miracidia were recorded by pen onto FTA card covers. These data were subsequently entered into a secure Microsoft Excel worksheet.
Timing and spatial scale	The fieldwork was conducted between 19/05/2014 and 14/06/2014. Sampling occurred at three schools within Mayuge district approximately 0.5 km from the Lake Victoria shoreline (Bugoto (0.32369,33.62837), Bwondha (0.17775,33.56138), Musubi (0.31105,33.6652) and one school within Tororo district (Kocoge (0.77552,34.23175)) located approximately 100 km from Mayuge district. These two districts, Mayuge and Tororo, were chosen as they had undergone long- and short-term MDA pressure, respectively. For all sites, approximately six months had elapsed between a prior round of MDA and the pre-treatment sample collection for this study.<br><br>Miracidia were isolated from infected children 1-3 days prior to treatment with praziquantel (40 mg/kg) for schistosomiasis and albendazole (400 mg) for soil-transmitted helminths. For cases of incomplete parasite clearance, post-treatment sampling was conducted a second time 25-27 days following treatment. This 25-27 day delay was chosen to allow enough time for parasite clearance following treatment but to prevent sampling parasites derived from reinfecting schistosomes, which would take 5-7 weeks to reach maturity (Colley et al. 2014).

Data exclusions	The original inclusion criteria (during the original survey by Crellen et al. (2016)) for efficacy analysis required that children be present for at least 1 day before and 1 day after treatment, they had to be positive for S. mansoni (based on at least 2 Kato-Katz thick smears) and they had to be successfully treated with praziquantel and albendazole without vomiting. Any child who was unwell (e.g. fever) did not take place in the study and were referred instead to the nearest local health unit.<br><br>Twenty four samples were excluded during variant calling quality control, we excluded samples with high rates of missing genotype calls (>55% of sites with a missing genotype) and/or excessively low inbreeding coefficient (< -0.3). In total, we excluded 23 samples from the Mayuge district (Bugoto n = 11, Bwondha n = 7, Musubi n = 5) and a single sample from 1 the Kocoge school.
Reproducibility	While multiple samples were taken from individuals, due to limited sample sizes all samples were included in the analysis. No replication was performed.
Randomization	From each infected child, all miracida hatched (up to 60 miracidia) were stored on Whatman FTA cards. For genomic analyses, DNA was obtained from a random five per card to control for any potential variance in hatching order.<br><br>Miracidia were allocated into groups based on sampling site (schools, districts), egg reduction rates (per child) and sampling time point (pre- or post-treatment).
Blinding	During sampling the investigators were not blinded to any aspect of the data. Blinding was not desirable or possible as i) no intervention or randomised treatment was imposed by the study, and ii) data were collected at the level of schools, which were chosen for inclusion based on their historical treatment history. Therefore key variables were known to the investigators a priori and the analysis explicitly aimed to detect differences in population genomic parameters arising from the past exposure to anthelmintic treatment. During genomic analyses researchers were blinded to all clinical data not required for group partitioning (examples of required for analyses include school, age, sex and egg reduction rates, pre-/post-treatment sampling).

Did the study involve field work? ☐ Yes ☒ No

Reporting for specific materials, systems and methods

We require information from authors about some types of materials, experimental systems and methods used in many studies. Here, indicate whether each material, system or method listed is relevant to your study. If you are not sure if a list item applies to your research, read the appropriate section before selecting a response.

Materials & experimental systems

n/a	Involved in the study
☒	☐ Antibodies
☒	☐ Eukaryotic cell lines
☒	☐ Palaeontology and archaeology
☒	☐ Animals and other organisms
☐	☒ Human research participants
☒	☐ Clinical data
☒	☐ Dual use research of concern

Methods

n/a	Involved in the study
☒	☐ ChIP-seq
☒	☐ Flow cytometry
☒	☐ MRI-based neuroimaging

Human research participants

Policy information about studies involving human research participants

Population characteristics	Children were aged 6-12 years, there were 15 female children and 19 male children (Supplementary table 1). Schools were selected based on mass drug administration histories: 1 round for Kocoge; 8 rounds for Musubi and 9 rounds for Bugoto and Bwondha, which we classify as short and long term high previous exposure to MDA. For each site at least six months had passed between the previous round of MDA and pre-treatment sample collection for this study. Children were followed up 25–27 days after treatment, at a point which would identify any treatment failures but not reinfections.
Recruitment	Within Uganda, districts, sub-counties and schools were contacted about the nature of the fieldwork by the Head of the Vector Control Division, Ministry of Health Uganda. The head-teacher of the school was informed fully about the study and requested to provide informed consent, allowing the field-teams to collect samples from children within the school. Parents of children at the school were informed of the study through school meetings and were requested to provide informed consent for their children to participate within the study. Prior to consent they were provided with detailed information as to why the study is taking place and any questions were answered by the authors and technical staff that were providing the information for the meeting. In addition to this, any child included who had reached age 10, they were also asked to sign and give informed consent after receiving full information of the study. From those children from which their parents have provided informed consent, random selection was undertaken by the field-teams. Participation was voluntary, and children could withdraw or be withdrawn from the study at any time. Access to treatment was not dependent on consenting to participate in the study. All infected children were provided with praziquantel at 40mg/kg.
Ethics oversight	This study was undertaken as part of monitoring and evaluation research activities conducted by the Schistosomiasis Control Initiative, Imperial College London and the Vector Control Division of the Ministry of Health, Uganda inherent with ongoing national disease control programme activities. All methods were approved by the Uganda National Council for Science and Technology (Memorandum of Understanding: sections 1.4, 1.5, 1.6) and the Imperial College Research Ethics Committee (EC NO: 03.36. R&D No: 03/SB/033E).

Note that full information on the approval of the study protocol must also be provided in the manuscript.
